# Detection, signal processing, and calibration In lmmunoassay systems

**DOI:** 10.1155/S146392469100010X

**Published:** 1991

**Authors:** P. A. Bonini, G. Banfi, M. Pazzagli, G. Messeri, A. Roda

**Affiliations:** 1 Istituto H.S. Raffaele Dipartimento di Medicina di Laboratorio Milano 20132 Italy; 2 Universita' di Firenze Istituto di Endocrinologia Firenze Italy; 3 Ospedale di Careggi Laboratorio Analisi Firenze Italy; 4 Università di Messina Istituto di Chimica Messina Italy

## Abstract

The new trends in immunochemistry related to the replacement of radioisotopic labels with non-radioactive labels are presented. Immunoenzymatic, fluorescent and chemiluminescent techniques are described in terms of their basic principles and their most common applications. The advantages of computer-controlled calibration are also discussed.


Journal of Automatic Chemistry, Vol. 13, No. 2 (March–April 1991), pp. 45–47

### Detection, signal processing, and calibration in immunoassay systems

**P. A. Bonini, G. Banfi**

*Istituto H.S. Raffaele, Dipartimento di Medicina di Laboratorio, 20132 Milano, Italy*

**M. Pazzagli**
*Universita' di Firenze, Istituto di Endocrinologia, Firenze, Italy*

**G. Messeri**
*Ospedale di Careggi, Laboratorio Analisi, Firenze, Italy*

**and A. Roda**
*Università di Messina, Istituto di Chimica, Messina, Italy*

*The new trends in immunochemistry related to the replacement of radioisotopic labels with non-radioactive labels are presented. Immunoenzymatic, fluorescent and chemiluminescent techniques are described in terms of their basic principles and their most common applications. The advantages of computer-controlled calibration are also discussed.*

### Introduction

The problems of signal detection and elaboration in nonisotopic assays are clearly related to the kind of label used. Various physical properties (other than radioactivity) of the label itself, or, alternatively, of label-mediated products are measured to monitor immunochemical reactions.

The signal can be detected either continuously or in a non-continuous way. An example of the first case is the measurement of the optical density of an enzyme mediated reaction; photon counting during fluorescent or chemiluminescent reactions is typical of non-continuous detection. Radioisotopes, which have been the most popular labels for immunochemical reactions in the last 30 years, are increasingly being replaced by nonisotopic labels; nevertheless, radioisotopes are the 'gold standards' when comparing the performances (sensitivity and specificity) of new labels.

### Specific activity and detection limits

The term 'specific activity' was used in the past to indicate the number of radioactive disintegrations per unit of time per unit of weight of the isotope compound in radioisotopic labelled immunoassays. It is now used, in a wider sense, to indicate detectable events per unit of time per unit of weight of label material in nonisotopic mediated reactions. As shown in table 1, the specific activity of chemiluminescent and fluorescent labels is notably higher than with radioisotopes [1]. This does not mean that chemiluminescent of fluorescent labels always result in more sensitive tests: in fact, many factors play an important role in determining sensitivity: the most important one is the possible steric hindrance due to some

*Table 1. Activities of isotopic and nonisotopic labels.*

| | |
|---|---|
| $^{125}I$ | 1 detectable events/s/7·5 × $10^6$ labelled molecules |
| $^{3}H$ | 1 detectable event/s/5·6 × $10^8$ labelled molecules |
| Enzymes | Determined by detectability of reaction product |
| Chemiluminescent labels | 1 detectable event/s/labelled molecule |
| Fluorescent labels | Many detectable events/s/labelled molecule |

labelling substances in contact between antigens and antibodies. Consequently, various isotope and nonisotope mediated immunochemical reactions show very wide ranges of sensitivity: table 2 shows the best detection limits reported in literature under a variety of conditions [2].

### Immunoenzymatic techniques

Many enzymes are widely used as labels in immunochemical reactions: table 3 shows the characteristics of an ideal enzyme label [3]. Peroxidase (HRP) (EC 1.11.1.7), alkaline phosphatase (ALP) (EC 3.1.3.1), and beta-galactosidase (Gal) (EC 3.2.1.23) are the enzymes most commonly used in immunochemistry; acetylcholinesterase (EC 3.1.1.7), glucose-6-phosphatedehydrogenase (EC 1.1.1.49), catalase (EC 1.11.1.6), and other enzymes are less frequently used. ALP usually splits paranitrophenylphosphate (pNPP) into phosphate and photometrically measurable paranitrophenol, but also methylumbelliferylphosphate can be used with production of fluorescent methylumbelliferone. Orthonitrophenylgalactopyranoside (oNPG), as well as methylumbellipherylgalactoside, can be used as substrates of Gal with a final photometric or fluorimetric reaction. The availability of different substrates for a single enzyme (for example for peroxidase) can result in the release of different enzymatic products with various absorbance spectra: this can facilitate the automation of such reactions [4].

Stopping enzymatic reactions by using various solutions (table 4) can be very useful in manual determinations, even if potentially harmful substances need to be used; in automatic systems this step can be avoided.

Enzymes are, *per se*, able to amplify the immunochemical reactions; their amplifying power can be further enhanced as shown in figure 1. The left-hand side of the figure shows the labelling enzymes (aldolase or phosphoglucoisomerase) acting as primary system enzymes in an amplifying system, and generating,

0142–0453/91 $3.00 © 1991 Taylor & Francis Ltd.

*Table 2. Detection limits of different labels used in immunoassays (reprinted by permission of T. Luider [Editor] from 'Thermochemiluminescence and its Application in Immunoassay' [Drukkers, Groningen, 1988]).*

| Type of label | Examples of labels | Detection limit |
|---|---|---|
| Radio-isotopes | $^{3}H$ | $5 \times 10^{-15}$ mole |
| | $^{125}I$ | $5 \times 10^{-17}$ mole |
| Enzymes | Beta-galactosidase | $1{\cdot}5 \times 10^{-16}$ |
| | Horseradish peroxidase | $3 \times 10^{-16}$ mole |
| | Alkaline phosphatase | $5 \times 10^{-17}$ mole |
| Bioluminescence | Firefly luciferin/luciferase | $5{\cdot}6 \times 10^{16}$ photons/min/mg |
| | Enzyme enhanced | $10^{-19}$ mole |
| | Firefly luciferin/luciferase | $10^{-19}$ mole |
| Fluorescence | Europium(III) ion | $2 \times 10^{-17}$ mole |
| | Fluorescein | $10^{-13}$ mole |
| Enzymes/chemiluminescence | Peroxidase/luminol/enhancer | $<10^{-15}$ mole |
| | Glucose-6-phosphate dehydrogenase peroxidase/ isoluminol | $10^{-18}$ mole |

respectively, fructose-bisphosphate (FBP) from glyceraldehydetriphosphate (G3P) + dihydroacetonphosphate (DHAP) and fructose-6-phosphate (F6P) from glucose-6-phosphate (G6P). Either FBP or F6P can act as substrates for the secondary system, driven by the enzymes phosphofructokinase and fructosebisphosphatase, with production of inorganic phosphate. Redox cycle is a secondary amplifying system as shown in the right-hand side of the figure: the labelling enzyme ALP, acting as primary system enzyme, generates the substrate nicotinamide adeninnucleotide (NAD) for the redox cycle driven by the enzymes alcoholdehydrogenase and lipoamidedehydrogenase; the cycle needs an excess of ethanol and generates formazan dye as product. A ten-fold increase in absorbance can be obtained in this way [5].

Nonisotopic methods based on photon counting as signal measurement include fluorescence and chemiluminescence.

### Fluorescent and chemiluminescent techniques

In fluorescence a molecule (fluorophor), excited by light, moves to an excited state and then returns to the natural state. Energy is emitted during this process as photons and then measured without chemical modification. Conversely, chemiluminescence is based on a chemical transformation of a substance into another one. In such a chemical reaction, energy is emitted and then measured. In the field of fluorescent immunoassays (FIA), various methods have been implemented and are routinely used; the most popular are described below.

*Fluorescence polarization immunoassay* (FPIA) is characterized by a fluorogenic label being excited by polarized light and emitting a partially polarized fluorescence at a right angle to the incident beam.

*Front surface fluorescence* is characterized by a fluorescence mediated immunoreaction taking place on a solid-phase device, reflecting the generated fluorescence in the same direction as the exciting light.

*In time resolved fluorescent immunoassays* (TRF) immunoreagents are labelled with a special class of fluorogens (lanthanide ions, usually Europium), characterized by emission peaks at 614 nm, very narrow and well separated from the scattering caused by excitation (at 340 nm) and from interfering fluorescence of serum (from 400 to 600 nm) (see figure 2). In addition, the specificity of this technology is further enhanced by the decay time of these substances, which is much longer (10–1000 µs) than that of the background (1–20 ns); thus, specific fluorescence can be detected once the interfering one has fully decayed [6].

*Luminescence* is based on the property of some natural (bioluminescence) or non-natural (chemiluminescence) chemical compounds to emit energy (photons) when

*Table 3. Characteristics of an ideal enzyme label.*

| |
|---|
| High enzyme activity at low substrate concentration |
| Enzyme stable at pH required for good antibody–antigen binding |
| Presence of reactive groups through which enzymes can be covalently linked to antibody, antigen or hapten with minimum loss of enzyme or immune activities |
| Availability of soluble, purified enzyme at low cost |
| Absence of health hazards attributable to enzyme, substrates and cofactors |
| Enzyme labelled conjugates stable under routine storage and assay conditions |

*Table 4. Stopping solutions currently used in immunochemistry.*

| | | |
|---|---|---|
| HRP | OPD → | sulphuric acid 2 mol/l |
| | ABTS → | sodium azide 10 mmol/l |
| | oDIA → | hydrochloric acid 5 mol/l |
| | TMB → | sulphuric acid 1 mol/l |
| ALP | pNPP → | sodium carbonate 0·5 mol/l |
| Beta gal | oNPG → | sodium carbonate 0·5 mol/l |

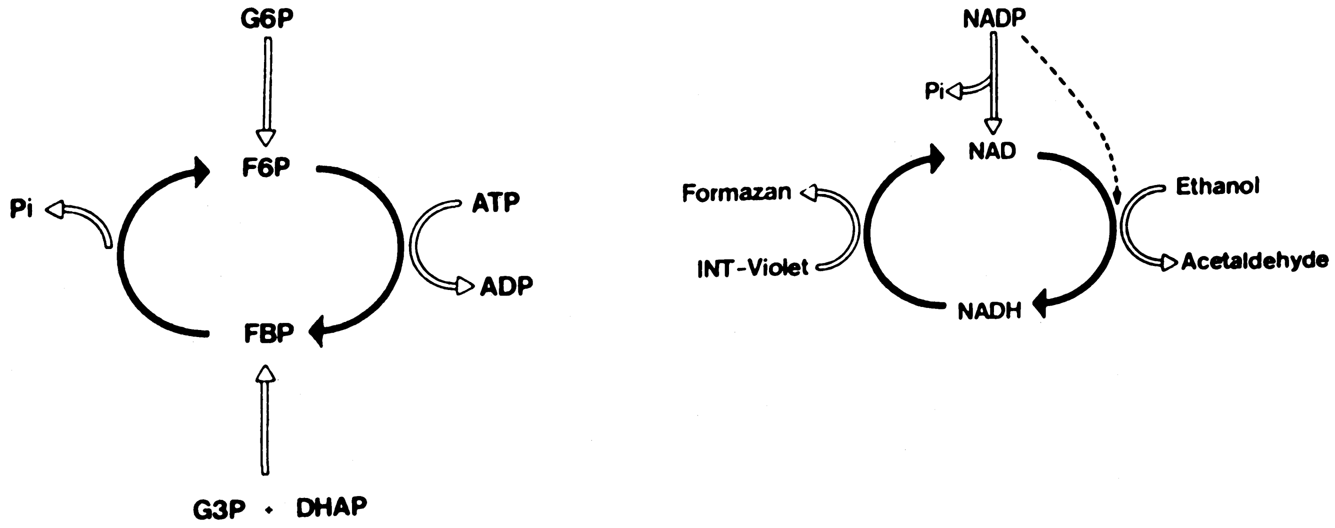

*Figure 1. Amplification of enzyme immunoassays.*

enzymatically oxidized. The most commonly used molecules in chemiluminescence are isoluminol and acridinium esters. Some chemical compounds (typically phenol and naphthols) are used to enhance chemiluminescence—this results in a raised intensity of the emitted light, as well as in its transformation from a flash to a constant glow. A 1000 times amplification and a stabilization up to 20 minutes of the signal is obtained in this way, thus allowing an optimization of the reaction [7].

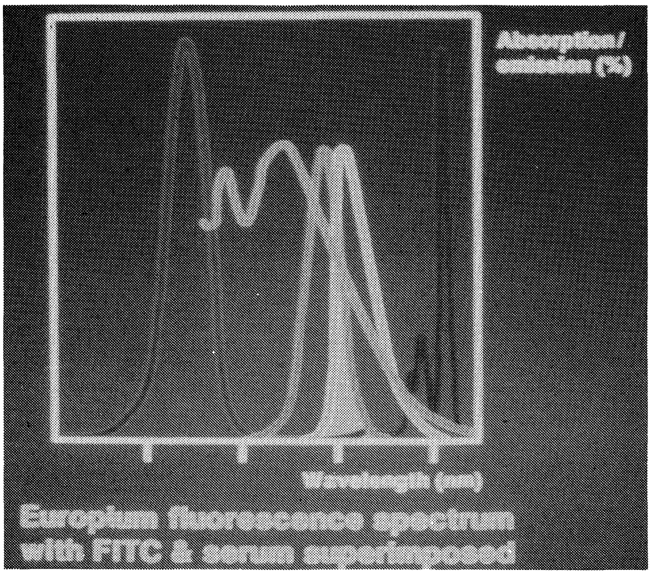

*Figure 2. Time resolved fluoroimmunoassays. From left to right: excitation spectrum of Europium, emission spectrum of serum, excitation and emission spectra of fluorescein, and absorption spectrum of Europium.*

### Calibration

Calibration is an important step in an immunochemical reaction: it is classically performed by using various standards, containing different, well-defined amounts of the analyte. This calibration, typical of the manual procedures, has been applied to semi-automated and automated instruments. The introduction of automation to immunochemistry has meant that the calibration points in the daily work have been reduced to one or two points, while the complete multipoint standard curve is stored in the computer. The advantages of such procedure are obvious.

A very simple and inexpensive calibration system is represented by so-called 'master curve': the 'true' calibration in these cases is performed by the manufacturer and memorized by the computer which is then able to retrieve the calibration data following an instruction from the operator (usually represented by a barcode connected to a single lot of reagents).

### References

1. Ekins, R., Chu, F. and Micaleff, J., In *Alternative Immunoassays* (John Wiley and Sons, New York, 1985), 64–66.
2. Luider, T. (Ed.), *Thermochemiluminescence and its Application in Immunoassay* (Drukkers, Groningen, 1988), 23–24.
3. Blake, C. and Gould, B. J., *Analyst,* **109** (1984), 533.
4. Alkaissi, E. and Mostratos, A., *Journal of Immunological Methods,* **58** (1983), 127.
5. Bates, L. D., *Annales de Biologie Clinique,* **47** (1989), 527.
6. Soini, E. and Hemmila, I., *Clinical Chemistry,* **25** (1979), 353.
7. Pazzagli, M. and Messeri, G., In *Non Radiometric Assays: Technology and Application in Polypeptide and Steroid Hormone Detection* (Alan R. Liss, New York, 1988), 61–77.

